# No Evidence for Mutations of *CTCFL*/*BORIS* in Silver-Russell Syndrome Patients with *IGF2/H19* Imprinting Control Region 1 Hypomethylation

**DOI:** 10.1371/journal.pone.0006631

**Published:** 2009-08-13

**Authors:** Jeremiah Bernier-Latmani, Alessandra Baumer, Phillip Shaw

**Affiliations:** 1 Experimental Pathology Division, Institute of Pathology, University of Lausanne, Lausanne, Switzerland; 2 Institute of Medical Genetics, University of Zürich, Schwerzenbach, Switzerland; Stanford University, United States of America

## Abstract

**Background:**

Silver-Russell syndrome (SRS) is a genetically and clinically heterogeneous disease. Although no protein coding gene defects have been reported in SRS patients, approximately 50% of SRS patients carry epimutations (hypomethylation) at the *IGF2/H19* imprinting control region 1 (ICR1). Proper methylation at ICR1 is crucial for the imprinted expression of *IGF2*, a fetal growth factor. CTCFL, a testis-specific protein, has recently been proposed to play a role in the establishment of DNA methylation at the murine equivalent of ICR1. A screen was undertaken to assess whether *CTCFL* is mutated in SRS patients with hypomethylation, to explore a link between the observed epimutations and a genetic cause of the disease.

**Methodology/Principal Findings:**

DNA was obtained from 36 SRS patients with hypomethylation at ICR1. All *CTCFL* coding exons were sequenced and analyzed for duplications/deletions using both multiplex ligation-dependent probe amplification, with a custom *CTCFL* probe set, and genomic qPCR. Novel SNP alleles were analyzed for potential differential splicing *in vitro* utilizing a splicing assay. Neither mutations of *CTCFL* nor duplications/deletions were observed. Five novel SNPs were identified and have been submitted to dbSNP. *In silico* splice prediction suggested one novel SNP, IVS2-66A>C, activated a cryptic splice site, resulting in aberrant splicing and premature termination. *In vitro* splicing assays did not confirm predicted aberrant splicing.

**Conclusions/Significance:**

As no mutations were detected at *CTCFL* in the patients examined, we conclude that genetic alterations of *CTCFL* are not responsible for the SRS hypomethylation. We suggest that analysis of other genes involved in the establishment of DNA methylation at imprinted genes, such as DNMT3A and DNMT3L, may provide insight into the genetic cause of hypomethylation in SRS patients.

## Introduction

Silver-Russell syndrome (SRS) is a rare and genetically heterogeneous disease (OMIM: 180860). Diagnosis of SRS includes: low birth weight and height, poor postnatal growth, skeletal asymmetry, triangular facial features and distinct head shape [1]. The etiology of the disease remains elusive as no protein coding gene mutations have been identified, although maternal uniparental disomy of chromosome 7 is observed in ∼10% of SRS patients [1]. More recently, however, an epimutation, hypomethylation of the *IGF2/H19* imprinting control region 1 (ICR1) at 11p15, was observed in SRS patients and is now reported in approximately 50% of cases [1], [2]. Moreover, the extent of hypomethylation at ICR1 has recently been correlated to the severity of the disease [3], [4].

Methylation of the paternal ICR1 is crucial for imprinted expression of the two adjacent genes, *IGF2* and *H19*. *IGF2* codes for a fetal growth factor and is expressed uniquely from the paternal allele, while *H19*, a non-coding RNA, is expressed solely from the maternal allele [5], [6]. ICR1 is unmethylated on the maternal allele which allows binding by the insulator protein CTCF. CTCF blocks enhancer access to the *IGF2* promoter, resulting in the silencing of *IGF2* on the maternal allele [7]. However, methylation of the paternal ICR1 abrogates CTCF binding and *IGF2* expression is activated [8], [9]. Diminished *IGF2* expression, through ICR1 hypomethylation and subsequent CTCF binding and *IGF2* enhancer blocking on the paternal allele, is thought to be responsible for the low birth weight and poor post-natal growth observed in SRS patients. Therefore, the ICR1 hypomethylation epimutation provides the strongest insight into the genetic cause of SRS and suggests that gene products involved in the establishment of DNA methylation at ICR1 may be mutated in SRS patients with hypomethylation.

A mechanism for the establishment of DNA methylation at murine imprinted genes has recently been proposed involving the protein CTCFL/BORIS [10]. CTCF-like (CTCFL) or Brother Of the Regulator of Imprinted Sites (BORIS), hereafter called CTCFL, and the ubiquitously expressed CTCF are closely related by 79% similarity among the 11 zinc fingers they both contain [11]. However, *CTCFL* is uniquely expressed in the testis and shares no significant similarity in either the N- or C-termini to CTCF, suggesting that the two proteins perform different functions, although they most likely bind similar DNA sites [11]. Our laboratory has shown that CTCFL binds the murine equivalent of ICR1, the *Igf2/H19* ICR, *in vivo* and interacts with the arginine methyltransferase PRMT7 and histones H1, H2A and H3. PRMT7 methylates histones H2A and H4 and CTCFL stimulates PRMT7-mediated histone methylation. Additionally, when CTCFL is expressed in *Xenopus* oocytes, with PRMT7 and the *de novo* DNA methyltransferases 3A, 3B and L (DNMT3A, B, L), which are essential for the establishment of methylation at imprinted genes [12]–[14], CpG dinucleotides of a plasmid containing murine ICR1 are methylated [10]. The current model contends that CTCFL specifically binds the *Igf2/H19* ICR, recruits PRMT7, which then methylates nearby histones. This histone methylation can then serve as a recruitment signal for the *de novo* DNA methyltransferases which methylate the CpGs of the *Igf2/H19* ICR. Recently, DNMT3A recruitment mediated by PRMT5 histone arginine methylation has been demonstrated, consistent with the proposed model [15].

Based on observations of hypomethylation at ICR1 in SRS patients and the proposed role of CTCFL in directing DNA methylation at the *Igf2/H19* ICR, we hypothesized that SRS patients with hypomethylation at ICR1 could potentially harbor mutations in *CTCFL*, which would provide a genetic link to the epimutations observed in these patients. To test this hypothesis, 36 SRS patients with hypomethylation at ICR1 were screened for mutations in *CTCFL*. Additionally, the SRS patients were screened for exonic duplications/deletions using multiplex ligation-dependent probe amplification (MLPA) and qPCR. Lastly, a novel SNP revealed by *CTCFL* sequencing, and predicted *in silico* to activate a cryptic splice site, was tested for possible alternative splicing.

## Results

### Sequence analysis

**Figure 1:**
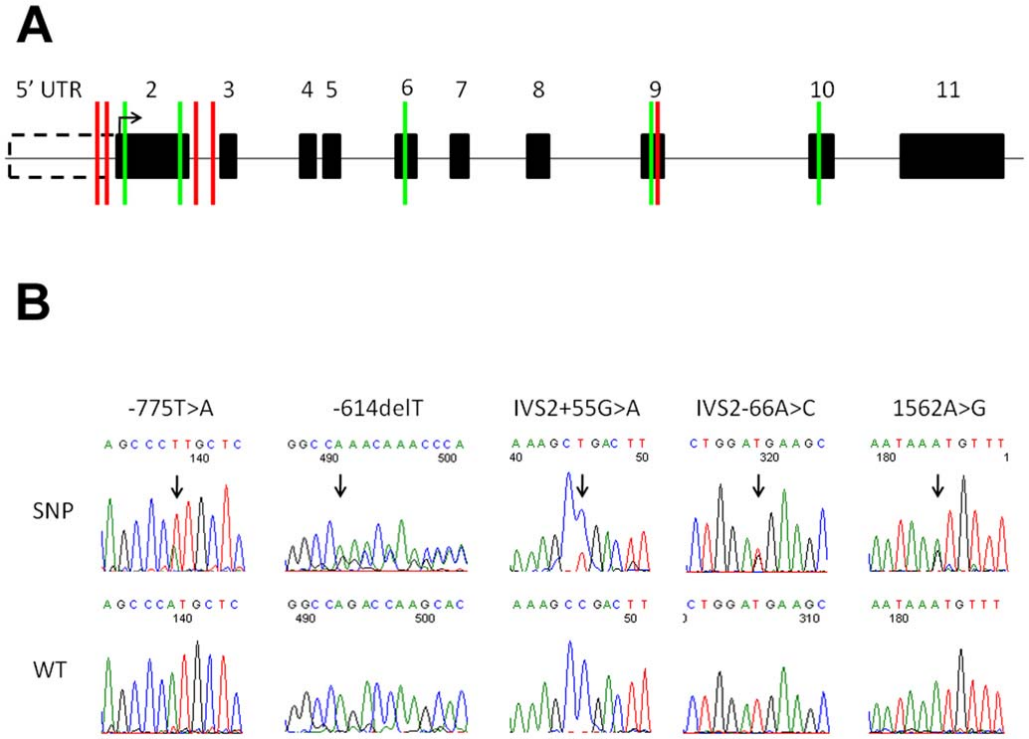
Distribution of polymorphic HapMap and novel SNPs within *CTCFL*. A) *CTCFL* gene structure showing relative positions of exons (solid boxes) and 5′UTR (dashed box). Sequencing of SRS patient DNAs led to the detection of novel SNPs (red dashes) and polymorphic HapMap SNPs (green dashes). B) Representative chromatograms of novel SNPs in comparison to wild-type sequence.

*CTCFL* consists of 10 coding exons and 3 alternative first exons, which will be denoted here as the 5′UTR (Figure 1A) [11], [16]. All coding exons and the 5′UTR of *CTCFL* were sequenced in 36 SRS patients with hypomethylation at ICR1. Sequencing revealed SNPs present in dbSNP and included 5 polymorphic HapMap SNPs (Figure 1A). The HapMap SNPs allele frequencies did not significantly differ between SRS patients and the CEU population (Table 1). Five novel SNPs (not listed in either dbSNP or ABI SNP databases) were found in *CTCFL* among the SRS patients (Figure 1A, 1B), but the frequency of the observed SNPs did not significantly differ between the patients and healthy controls (Table 2). All novel SNPs have been deposited in dbSNP (Table 2). No nonsense or missense mutations were found in any of the patients, but one heterozygous silent mutation (1562A>G; K>K) was observed in exon 9 of one patient (Figure 1B).

**Table 1:** Polymorphic HapMap SNPs at *CTCFL* in SRS patients and CEU population.

Exon	Genotype	Frequency in SRS Patients	Hapmap Frequency (CEU)	dbSNP ID
2 (5′)	C/C	18 (50%)	30.0%	rs6070128
	C/G	12 (33.3%)	56.7%	
	G/G	6 (16.7%)	13.3%	
2 (3′)	C/C	10 (27.8%)	38.3%	rs6025606
	C/T	18 (50%)	51.7%	
	T/T	8 (22.2%)	10%	
6	A/A	26 (72.2%)	72.4%	rs6025601
	A/G	10 (27.8%)	24.1%	
	G/G	0 (0%)	3.4%	
9	G/G	35 (97.2%)	90%	rs6070122
	G/C	1 (2.8%)	10%	
10	G/G	20 (55.6%)	51.7%	rs6128059
	G/A	13 (36.1%)	41.7%	
	A/A	3 (8.3%)	6.6%	

**Table 2:** Novel SNPs and frequencies in SRS patients and healthy controls at *CTCFL*.

SNP	Genotypes	Frequency in patients	Frequency in controls	dbSNP ID
-775T>A	T/T	20 (87%)	42 (97.7%)	ss115492397
	T/A	3 (13%)	1 (2.3%)	
-614delT	T/T	21 (91.3%)	41 (91.1%)	ss115492398
	del/T	2 (8.7%)	4 (8.9%)	
IVS2+55G>A	G/G	35 (97.2%)	95 (100%)	ss115492399
	G/A	1 (2.8%)	0	
IVS2-66A>C	A/A	32 (88.9%)	95 (95%)	ss115492400
	A/C	4 (11.1%)	5 (5%)	
1562A>G	A/A	35 (97.2%)	95 (100%)	ss115492401
	A/G	1 (2.8%)	0	

### Exon duplication/deletion analysis

**Figure 2:**
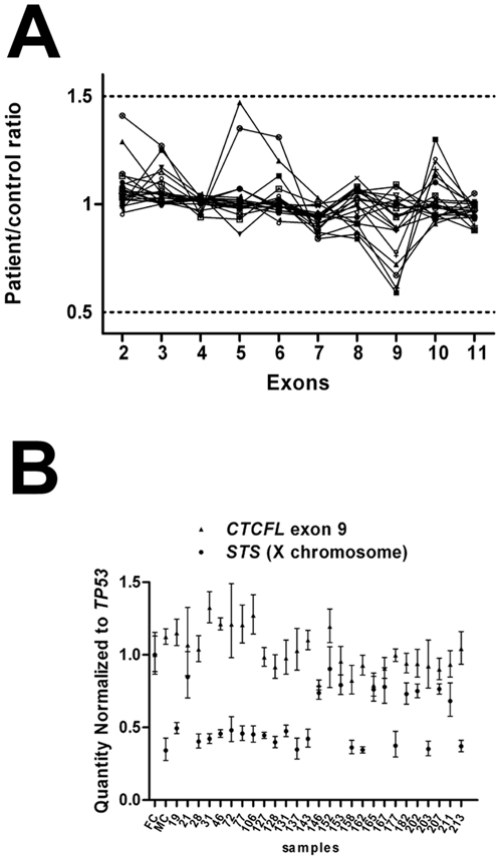
*CTCFL* exon duplication/deletion analysis in SRS patients. A) MLPA analysis of *CTCFL* exons in SRS patient DNA. Connected symbols represent individual patients. Values obtained relative to control samples are presented. B) Copy number analysis of *CTCFL* exon 9 by genomic qPCR. Each point represents the mean C_t_ for the given reaction, normalized by the mean C_t_ obtained for *TP53* (autosomal, two copies) from the same sample. All reactions were performed in quadruplicate and error bars represent standard error from the mean. All samples use FC (female control, non-SRS) as the reference sample. Numbers 19–213 refer to SRS patients and MC represents the male control (non-SRS).

*CTCFL* was also screened for exonic deletions or duplications in these same patients using MLPA. Custom oligonucleotide MLPA probes were designed for each exon (Table S2). Twenty-one patients were screened at exons 2–11 and normalized by comparison to 7 healthy controls. Two SRS patients had a normalized value of 0.6 for exon 9 of *CTCFL*, suggesting a possible deletion (Figure 2A). To further examine exon 9 deletion in these patients, genomic qPCR was performed. *CTCFL* exon 9 was amplified in 27 patients and compared to levels of a diploid control, *TP53* (Figure 2B). Also, as a second control, qPCR was performed on the X chromosome gene *STS* which is single copy in males. No patient had a quantity of exon 9 as low as the male patients at the *STS* locus (Figure 2B). Moreover, the copy number of *CTCFL* exon 9 for patients with the possible deletion was not significantly different from *TP53* (Figure 2B), suggesting that exon 9 of *CTCFL* is not deleted in these patients.

**Figure 3:**
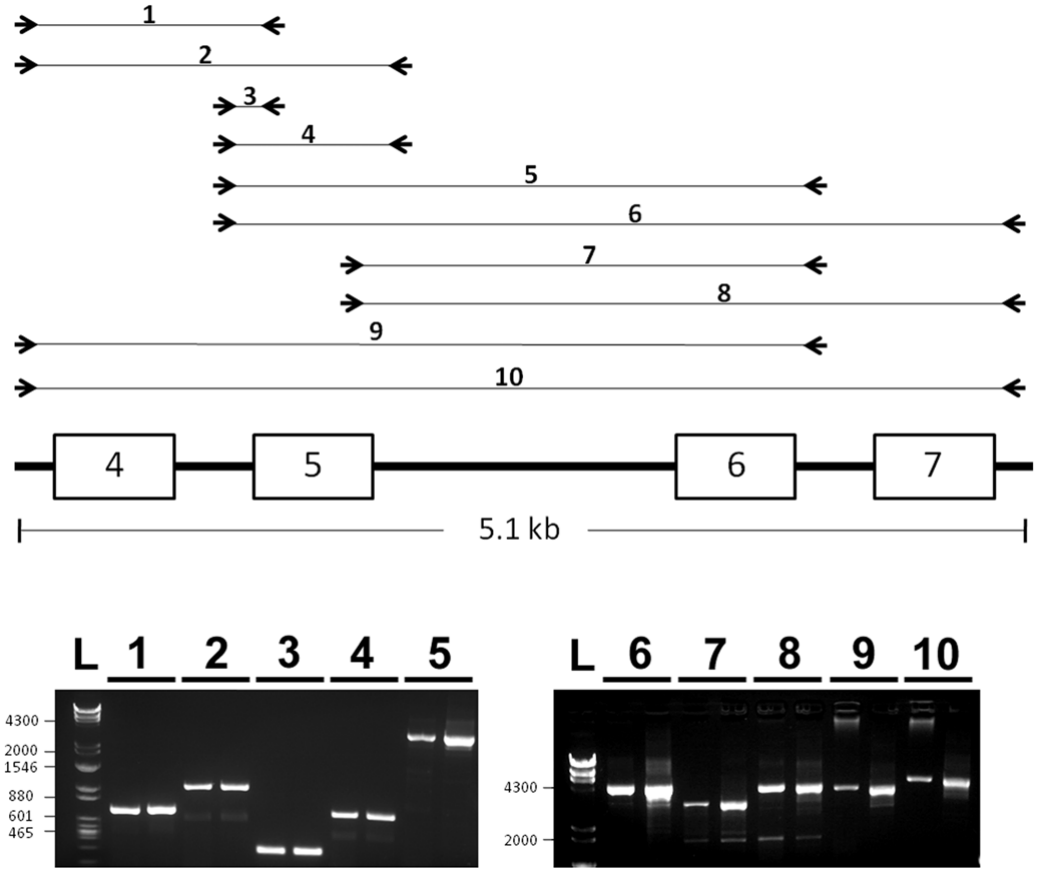
PCR analysis of exon 5 duplication. MLPA analysis suggested exon 5 duplication in SRS patient 162. Ten separate PCR reactions were performed to analyze exon 5 and adjacent genomic regions for evidence of duplication (scheme shown on top). PCR products from the respective reactions are shown for both patient 162 (left lane) and control (right lane) DNA. The sequences of primers used are given in Table S4.

MLPA also indicated a possible duplication of exon 5 in two patients with normalized ratios at or above 1.4 (Figure 2A). Extensive PCR analysis of exon 5 and the surrounding genomic region did not provide supporting evidence for exon 5 duplication (Figure 3).

### Splicing analysis

**Figure 4:**
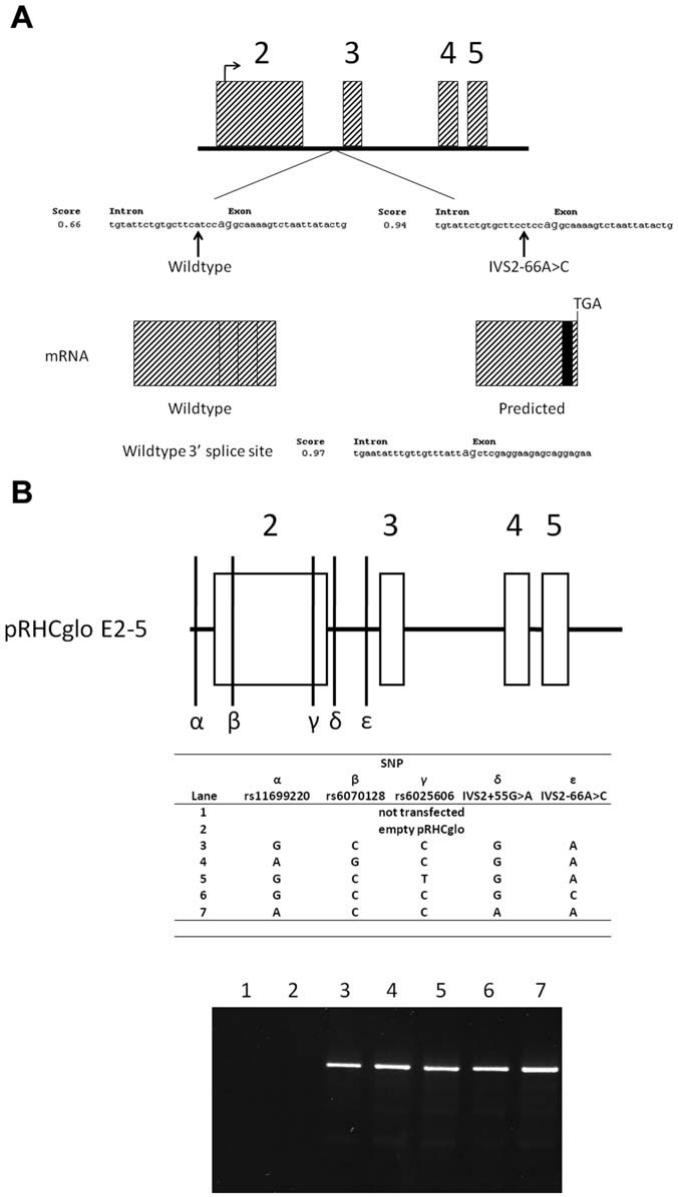
Splicing analysis of *CTCFL* exons 2 and 3. A) Expanded view of *CTCFL* exons 2–5 with sequence upstream of exon 3 with the location of SNPs indicated. The sequences and scores are outputs from the FlyBase Splice Prediction program. Higher scores indicate better alignment of the sequence to known splice sites. Arrows mark the nucleotide position changed by IVS2-66A>C and the score at the cryptic splice site increases from 0.66 to 0.94 with the single nucleotide substitution. Also shown is the wild-type *CTCFL* splicing according to the demarcated exon/intron junctions. Splicing for the IVS2-66A>C substitution is predicted to splice exon 2 to a cryptic splice site 61 bp upstream of the wild-type 5′exon 3 splice site (shown on bottom with the associated FlyBase score). Mis-splicing predicts a frame shift and premature termination in exon 3. B) The genomic region cloned into pRHCglo is shown and the relative positions of the SNPs analyzed are denoted α-ε. The table shows the haplotypes for each minigene cloned into pRHCglo which were derived from both SRS patient and control DNA. Each pRHCglo E2-5 plasmid was transfected into 293T cells, the RNA extracted and reverse transcribed. PCR products were run on an agarose gel and visualized with ethidium bromide. No alternative splice products were observed.

As four of the five novel SNPs were observed in introns, analysis was undertaken to determine if these novel SNPs could affect splicing of *CTCFL*. *In silico* prediction of splicing using sequence from both the wild-type and novel SNPs was performed using two online tools, Flybase Splice Site Predictor and ESE Finder. All novel SNPs were tested, but only one SNP, IVS2-66A>C, was predicted by both programs to activate an alternative 3′ splice site four nucleotides downstream from the SNP itself and to consequently add 61 bps to exon 3 resulting in a frame-shift and premature termination (Figure 4A). Testis samples from the SRS patients, where *CTCFL* is uniquely expressed, were not available [10], [11]. We therefore analyzed splicing among IVS2-66A>C and other nearby SNPs using a minigene splicing assay [17]. Total RNA was extracted from 293T cells transfected with a minigene plasmid (pRHCglo E2-5) containing the genomic region encompassing exons 2–5 of *CTCFL* with alleles carrying either the wild-type or variant SNP at IVS2-66. As other SNPs are located in the genomic region contained in pRHCglo E2-5, these were also tested for possible differential splicing. These SNPs included: two HapMap SNPs (rs6070128 and rs6025606), one other novel SNP (IVS2+55G>A) and a previously observed non-HapMap SNP (rs11699220) (Figure 4B). After specific reverse transcription and PCR of the spliced minigene transcript, no differences in splicing were observed for any of the alleles tested (Figure 4B).

## Discussion

All *CTCFL* exons were sequenced in SRS patients to test the hypothesis that mutations in this gene may be responsible for hypomethylation of ICR1. No missense or nonsense mutations were found. However, five novel SNPs were identified. As one of the novel SNPs (IVS2-66A>C) was predicted to activate a cryptic 3′ splice site near exon 3, a minigene splicing assay was used to determine if *CTCFL* undergoes SNP-dependent alternative splicing. Neither IVS2-66A>C nor alleles of nearby SNPs displayed alternative splicing at the exon 2/3 junction. These results rule out splicing aberrations of *CTCFL* as a cause of hypomethylation in these patients. Lastly, the exons of *CTCFL* in SRS patients were screened for duplications/deletions using MLPA and qPCR. No duplications/deletions were observed, strongly suggesting that genetic alterations of *CTCFL* are not present in these patients.

As maternal uniparental disomy of chromosome 7 is observed in ∼10% of SRS patients[1], previous SRS candidate gene studies have focused on chromosome 7 [18]–[24]. To our knowledge, this study is the first candidate gene approach to examine genes involved in the establishment of imprinted DNA methylation at ICR1. Our findings in this cohort of SRS patients do not indicate that mutations in *CTCFL* are a cause of the hypomethylation epimutation. If CTCFL participates in directing global imprinted gene DNA methylation as proposed [10], a mutation in the gene may lead to either an embryonic lethal or more severe phenotype than SRS. This is but one explanation for the absence of observed *CTCFL* alterations in SRS patients. Future investigation will clarify the full impact of CTCFL function on the establishment of DNA methylation during development.

Further investigation may also point to mutations in other genes/proteins participating in the establishment of DNA methylation at imprinted genes as a cause of hypomethylation in these patients. The *de novo* DNA methyltransferases DNMT3A and DNMT3L have been shown to be essential for normal imprinted DNA methylation [12], [13] and make strong candidates for a mutational screen in SRS patients with hypomethylation of ICR1 [25]. Lastly, a more comprehensive understanding of the mechanism of *de novo DNA* methylation at imprinted genes may provide novel candidate genes for further study into the cause of the hypomethylation epimutation and SRS.

## Materials and Methods

### Ethics Statement

The study was performed in accordance with the ethics review boards of the University of Zürich, University of Lausanne and the University Hospital of Lausanne (CHUV). Written informed consent for analysis of DNA was obtained from all adult patients and parents of underage patients included in this work.

### Patients

The study population consisted of a 36 patient subgroup from a pool of 201 SRS patients diagnosed by clinics in Zürich, Warsaw, Minsk, and Istanbul. These 36 patients were selected for further analysis due to hypomethylation at the *IGF2*/*H19* ICR as determined by methylation-specific MLPA [3]. The 36 patients have SRS severity scores ranging from 8 to 15 (mean, 11.8, 4 undetermined) as ascertained by Bartholdi et al [3]. One hundred unrelated healthy individuals served as controls. Genomic DNA was extracted as previously described [3].

### Sequencing

*CTCFL* genomic sequence was downloaded from NCBI Map Viewer and exons were demarcated using the NCBI cDNA sequence NM_080618.2. SNPs were identified using dbSNP and the ABI GeneAssist Genotyping Alignment Map (Applied Biosystems website). This map was then used to design intronic primers to individually amplify exons 2–11 and nested or partially nested primers sequencing primers to directly sequence the PCR products (Table S1). 5′UTR PCR primers were designed to encompass the entire 5′UTR as described by Renaud et al. [16] 100 ng of genomic DNA was used in 50 µl PCR reactions for each exon in each patient using either AmpliTaq Gold (ABI, exons 2–11) or Phusion DNA Polymerase (Finnzymes, 5′UTR). PCR reaction conditions are available upon request. After purification with the QIAquick PCR Purification Kit (Qiagen), PCR products were sequenced and run on an ABI 3130xl DNA Fragment Analyzer. Chromatograms were manually inspected using FinchTV (Geospiza).

### MLPA

MLPA reactions were performed using the SALSA MLPA kit (MRC-Holland, Amsterdam, Netherlands) according to manufacturer's instructions. Briefly, 100 ng of genomic DNA was denatured for 5 min. at 98°C and cooled to 25°C. A master mix containing the *CTCFL* probemix (Table S2), DQ- and DD-control probes (MRC-Holland) and MLPA buffer were added and the mixture was heated briefly (1 min.) to 95°C, before annealing the MLPA probes to the genomic DNA at 60°C overnight. A mastermix containing Ligase-65 Buffers A+B and Ligase-65 was added to the same tubes at 54°C and allowed to ligate for 15 min. followed by a 5 min. incubation at 98°C and stored at 4°C. 50 µl PCR reactions were performed using 10 µl of MLPA reaction product with SALSA PCR primers (FAM-labeled), enzyme dilution buffer and polymerase using the recommended PCR cycling conditions. The FAM-labeled PCR products were separated on an ABI 3130xl and the size was determined by the addition of ROX-250-labelled size standards (ABI). The MLPA PCR products were visualized with Peakscanner software (ABI) and quality-checked by the presence/absence of DQ- and DD-control fragments. The chromatograms were exported as .fsa files to Coffalyser (MRC-Holland) for statistical analysis. Chromatograms of 7 healthy controls were used to normalize those of SRS patients.

### qPCR

Genomic DNA from SRS patients and healthy controls was used for quantitative real-time PCR (qPCR) to screen for deletions at exon 8 and 9 of *CTCFL*. Primers were designed for exons 8 and 9 of *CTCFL*, exon 7 of *TP53* (diploid control) and exon 5 of steryl-sulfatase precursor (*STS*), an X chromosome gene (haploid control, in males) (Table S3).

This allowed normalization of all reactions to *TP53*, while facilitating analysis of possible deleted *CTCFL* exons by comparison to *STS* reactions in males, which have only one copy of the gene. The concentration for each primer pair was optimized for 10 µl reactions and was used as follows: *CTCFL* exon 8 200 nM, *CTCFL* exon 9 400 nM, *TP53* 200 nM, *STS* 600 nM. Reactions were performed using 50 ng of genomic DNA with 2X *Power* SYBR® Green PCR Master Mix (ABI), forward and reverse primer mix (5 mM) and water to 10 µl. All qPCR reactions were run on an ABI 7900HT using standard conditions.

### Minigene splicing assay

To analyze possible mis-splicng caused by novel SNPs in *CTCFL* we first used *in silico* methods to search for possible splice-altering SNPs. Two online programs, Flybase Splice Site Predictor (http://www.fruitfly.org:9005/seq_tools/splice.html)[26] and ESE Finder 3.0 (http://rulai.cshl.edu/cgi-bin/tools/ESE3/esefinder.cgi)[27] were used to analyze splice sites with or without the novel SNP and flanking sequence. To experimentally test for alternative splicing caused by novel SNPs we used a minigene splicing assay. 100 ng of patient or control DNA was used in PCR reactions with Phusion DNA polymerase to amplify the genomic region of *CTCFL* encompassing exons 2–5 (E2-5) using primers upstream of exon 2 (miniE2-5f: 5′- GCGGGATCCAGAGTGTGCTCAGGCGGAAC) and downstream of exon 5 (miniE2-5r: 5′- CGCACTAGTGTGAGTACCGCCAAACCTGTTAG). The PCR product was then digested with *Bam*HI and *Spe*I, gel-purified, and cloned into pRHCglo [17]. Individual colonies of DH10 transformed with pRHCglo E2-5 were picked, grown overnight and plasmid DNA was extracted using the QIAprep Spin Miniprep Kit (Qiagen). Plasmid DNA was sequenced to identify transformants of each allele. Next, 5 µg of pRHCglo E2-5 plasmid DNA was CaPO_4_-tranfected into 293T cells [28]. Cells were grown overnight, the media was changed the next morning and cells were left to grow for a total of 48 hours. Total RNA was extracted using TRI-Reagent (Sigma) and reverse transcription and PCR were performed using the SuperScript III One-Step RT-PCR System (Invitrogen). The primer TNIE4 (5′-AGGTGCTGCCGCCGGGCGGTGGCTG) was used for reverse transcription as described by Singh and Cooper [17]. PCR primers were designed and used to amplify the exon 2/3 boundary to evaluate splicing (splchkf: 5′- GTGTGGCCATTAGTATCCAG; splchkr: 5′- GCTGTAGGTTGATCCTCTTG). PCR products were then analyzed by agarose gel electrophoresis.

## Supporting Information

Table S1 Genomic CTCFL PCR and sequencing primers
(0.06 MB DOC)

Table S2 CTCFL MLPA Probes
(0.04 MB DOC)

Table S3 qPCR primers
(0.03 MB DOC)

Table S4 Exon 5 duplication PCR primers
(0.03 MB DOC)
