# Supplementary material for: No Evidence for Mutations of CTCFL/BORIS in Silver-Russell Syndrome Patients with IGF2/H19 Imprinting Control Region 1 Hypomethylation
Source: PLoS One. 2009 Aug 13;4(8):e6631. doi: 10.1371/journal.pone.0006631 (PMC2721151; doi:10.1371/journal.pone.0006631)
Supplement: Table S1 — Genomic CTCFL PCR and sequencing primers (0.06 MB DOC) [file pone.0006631.s001.doc]

Table S1: Genomic *CTCFL* PCR and sequencing primers

| PCR primers | |  |  |
| --- | --- | --- | --- |
| Exon |  | | Sequence |
| **5'UTR** | forward | | 5'-CCTGGTATCTCAGTGCCTCCTGTTG |
|  | reverse | | 5'-GCAGGCTTTGGAGTTGAAACAAGG |
| **2** | forward | | 5'-GGCCAGACCTTGTTTCAACTCC |
|  | reverse | | 5'-ATGCGCCTTACACTATTCCAATTAGC |
| **3** | forward | | 5'-TTCACTGCCACAACCCTGATG |
|  | reverse | | 5'-CAATTACCATCACCTGCCCATAAAG |
| **4** | forward | | 5'-ATCGGCCCATCTTGAAACAGC |
|  | reverse | | 5'-CTTAACCAGAAACATTCCAAGGAATTCATAG |
| **5** | forward | | 5'-AGGCTGTTTCTCTGGAATGATGTG |
|  | reverse | | 5'-TCTTGACCACAGATGGTTTATTTGTAATTTG |
|  | 3' for | | 5'-TGCAAGTATGCCAGTGTGGAG |
|  | 5' rev | | 5'-GTTACACTTGTAGGGCCTGGTTC |
| **6** | forward | | 5'-TGAAATATGCTCTGGTGCTCTGATG |
|  | reverse | | 5'-GTTGTGGCACAGTATCTATGCAGAC |
| **7** | forward | | 5'-TAAGCTGTCTGGGCTTGATTTGG |
|  | reverse | | 5'-AGGGCCAAGTTCCCGAAGAC |
| **8** | forward | | 5'-CCTGCCCAGGAAGGAACTAGC |
|  | reverse | | 5'-GAATGACTGGCTCACAGTTGCTTTC |
| **9** | forward | | 5'-GCTGCCTTGTTCAGAATGTGTTTG |
|  | reverse | | 5'-AGGCATGACAGATGCTCCTGAAG |
| **10** | forward | | 5'-GCCTGGCCTCCTATTACCTTCTTG |
|  | reverse | | 5'-TCACACTGACTTTCTCACGCTATGC |
| **11** | forward | | 5'-GAAGGCAGTAGTTGAGATCAGAATAGTACC |
|  | reverse | | 5'-ACTTGTGTCATCCATTGTCATGAACTTAATTG |
| Sequencing primers | | |  |
| **5'UTR** | forward | | 5'-GAGGGTGCTAGGTCTAGAAC |
| **5'UTR_1** | reverse | | 5'-CCCGCAGGAGGCACTGAGAG |
| **5'UTR_2** | reverse | | 5'-ACGGTTCTAGACCTAGCACCCTCAG |
| **2** | forward | | 5'-CAAAGCCTGCTAGGTTCC |
|  | reverse | | 5'-CCTGGGAAGTATTTGTACTG |
| **3** | forward | | 5'-TGCGAAATAATTAACATTGTG |
|  | reverse | | 5'-CATCACCTGCCCATAAAG |
| **4** | forward | | 5'-AACATGGTGTCCTGAATTG |
|  | reverse | | 5'-CACGATTCTACTGTAGAAAC |
| **5** | forward | | 5'-ATGCATCTGTGGTATTTATAG |
|  | reverse | | 5'-AGCCTGTTTGTAACAGATTC |
| **6** | forward | | 5'-AATGCTGATTAATGGAAGAC |
|  | reverse | | 5'-CAGTATCTATGCAGACTCTC |
| **7** | forward | | 5'-GGGCTTGATTTGGGAGTC |
|  | reverse | | 5'-GTACTTTGCAGGTTTATAGG |
| **8** | forward | | 5'-CCCGGTTTAGAGGAGAGG |
|  | reverse | | 5'-GTATCTTCAAGGTGGTAGAG |
| **9** | forward | | 5'-GAATGTGTTTGCAGTAATTC |
|  | reverse | | 5'-GAAGTCCTGGCAGGTTTC |
| **10** | forward | | 5'-GCCCTCGAAAGAACTCAG |
|  | reverse | | 5'-CGACTGGTGGACAAATAG |
| **11** | forward | | 5'-TGCCGTGTTCAAATTTAAG |
|  | reverse | | 5'-GGGCAGTGAACATGCAAC |
|  |  | |  |
