# Supplementary material for: No Evidence for Mutations of CTCFL/BORIS in Silver-Russell Syndrome Patients with IGF2/H19 Imprinting Control Region 1 Hypomethylation
Source: PLoS One. 2009 Aug 13;4(8):e6631. doi: 10.1371/journal.pone.0006631 (PMC2721151; doi:10.1371/journal.pone.0006631)
Supplement: Table S2 — CTCFL MLPA Probes (0.04 MB DOC) [file pone.0006631.s002.doc]

Table S2: *CTCFL* MLPA Probes

| Exon |  | MLPA Probes* | PCR Product  Size (bp) |
| --- | --- | --- | --- |
| **2** | Left | 5'-**GGGTTCCCTAAGGGTTGGA**CTGTCCTTTCTGAGCAATTCACCAAGATCAA | 104 |
|  | Right | 5'-AGAACTCGAGTTGATGCCGGAAAAAGGCCTG**TCTAGATTGGATCTTGCTGGCAC** |  |
| **3** | Left | 5'-**GGGTTCCCTAAGGGTTGGA**GTGGAAACAATGTCAGGAGATGAAAGA | 108 |
|  | Right | 5'-AGTGACGAAATTGTTCTCACAGTTTCAAATTCAAATGTG**TCTAGATTGGATCTTGCTGGCAC** |  |
| **4** | Left | 5'-**GGGTTCCCTAAGGGTTGGA**CTATTTCCTGCCTATAGGAGCAAAAGGAACCT | 112 |
|  | Right | 5'-TCCACTGTGATGTCTGCATGTTCACCTCTTCTAGAATG**TCTAGATTGGATCTTGCTGGCAC** |  |
| **5** | Left | 5'-**GGGTTCCCTAAGGGTTGGA**CAAGTGTAACGACTGCAACATGGCATTTGTCACCAGTGGAGA | 116 |
|  | Right | 5'-ACTCGTCCGACACAGGCGCTATAAACATACTC**TCTAGATTGGATCTTGCTGGCAC** |  |
| **6** | Left | 5'-**GGGTTCCCTAAGGGTTGGA**CTACAAGCTGAAACGCCACATGAGAACGCACTCAGGTA | 120 |
|  | Right | 5'-AGGGCTCTGGTGCTGAAGGCCTGATACCTACAGTGTTAAC**TCTAGATTGGATCTTGCTGGCAC** |  |
| **7** | Left | 5'-**GGGTTCCCTAAGGGTTGGA**CCAAATACCAGTGTCCCCATTGTGCCACCATCATTGCACGGA | 124 |
|  | Right | 5'-AAAGCGACCTACGTGAGTGGTTTAGCGATCTTTCTTTTAC**TCTAGATTGGATCTTGCTGGCAC** |  |
| **8** | Left | 5'-**GGGTTCCCTAAGGGTTGGA**GAAATGCCGCTACTGTTCTGCTGTCTTCCA | 100 |
|  | Right | 5'-TGAACGCTATGCCCTCATTCAGCACCAG**TCTAGATTGGATCTTGCTGGCAC** |  |
| **9** | Left | 5'-**GGGTTCCCTAAGGGTTGGA**GCTCCAAGTGTGGCAAAGGCTTTTCCCGCTGGGTAAGCTTACT | 132 |
|  | Right | 5'-TAAGTCACAGTAAATCCCCCATGGAAAGATCCCTCTATCTCAGGAGG**TCTAGATTGGATCTTGCTGGCAC** |  |
| **10** | Left | 5'-**GGGTTCCCTAAGGGTTGGA**CAGACATTCGGAGAAGTGTGGATCAGGGGAAGCAAAGTCGGCTGCT | 136 |
|  | Right | 5'-TCAGGAAAGGGAAGAAGAACAAGAAAGAGGAAGCAGACCATCCTGAAG**TCTAGATTGGATCTTGCTGGCAC** |  |
| **11** | Left | 5'-**GGGTTCCCTAAGGGTTGGA**GACCTGTGAAATGCTCCTCAACACGATGGATAAGTGAGAGGGATTCGGGT | 140 |
|  | Right | 5'-TGCGTGTTCACTGCCCCCAATTCCTAAAGCAAGTTAGAAGTTTTTAGC**TCTAGATTGGATCTTGCTGGCAC** |  |
|  |  | * Bold- MLPA PCR primer sequence, underlined- sequence homologous to *CTCFL* |  |
