# Supplementary material for: No Evidence for Mutations of CTCFL/BORIS in Silver-Russell Syndrome Patients with IGF2/H19 Imprinting Control Region 1 Hypomethylation
Source: PLoS One. 2009 Aug 13;4(8):e6631. doi: 10.1371/journal.pone.0006631 (PMC2721151; doi:10.1371/journal.pone.0006631)
Supplement: Table S3 — qPCR primers (0.03 MB DOC) [file pone.0006631.s003.doc]

Table S3: qPCR primers

| Target |  | Sequence |
| --- | --- | --- |
| ***CTCFL* Exon 9** | forward | 5'-ACATTCGTACCCACACTGGAGAG |
|  | reverse | 5'-CCTGAAGTGAGCGTTTAGAAGTTG |
| ***p53*** | forward | 5'-ACTGTACCACCATCCACTACAAC |
|  | reverse | 5'-GCTCCTGACCTGGAGTCTTC |
| ***STS*** | forward | 5'-TGACTTCTGTCACCACCCTTTAC |
|  | reverse | 5'-GGTGAAGACACTGCCCTCTC |
|  |  |  |
