# Supplementary material for: No Evidence for Mutations of CTCFL/BORIS in Silver-Russell Syndrome Patients with IGF2/H19 Imprinting Control Region 1 Hypomethylation
Source: PLoS One. 2009 Aug 13;4(8):e6631. doi: 10.1371/journal.pone.0006631 (PMC2721151; doi:10.1371/journal.pone.0006631)
Supplement: Table S4 — Exon 5 duplication PCR primers (0.03 MB DOC) [file pone.0006631.s004.doc]

Table S4: Exon 5 duplication PCR

| Reaction | Forward primer* | Reverse primer* | Predicted PCR product size (bps) |
| --- | --- | --- | --- |
| 1 | 4f | 5_5'rev | 717 |
| 2 | 4f | 5r | 930 |
| 3 | 5f | 5_5'rev | 323 |
| 4 | 5f | 5r | 692 |
| 5 | 5f | 6r | 3677 |
| 6 | 5f | 7r | 4722 |
| 7 | 5_3'for | 6r | 3264 |
| 8 | 5_3'for | 7r | 4309 |
| 9 | 4f | 6r | 4071 |
| 10 | 4f | 7r | 5116 |
| *From Table S1 |  |  |  |
